# The Diseases and Casualties of the Year 1799

**Published:** 1800-02

**Authors:** 


*The Diseases and Casualties of the Year* 1799.

| | |
|---|---|
| Abortive and Stillborn | 580 |
| Abscess | 27 |
| Aged | 1343 |
| Ague | 3 |
| Apoplexy and Suddenly | 249 |
| Asthma and Phthisick | 663 |
| Bedridden | 2 |
| Bleeding | 16 |
| Bursten and Rupture | 20 |
| Cancer | 48 |
| Childbed | 131 |
| Colds | 14 |
| Colic, Gripes, and Twisting of the Guts | 8 |
| Consumption | 4843 |
| Convulsions | 3794 |
| Cough, and Hooping Cough | 451 |
| Cramp | 1 |
| Croup | 16 |
| Diabetes | 1 |
| Dropsy | 906 |
| Ear-ach | 1 |
| Eaten by Lice | 1 |
| Evil | 5 |
| Fevers of all kinds | 1784 |
| Fistula | 3 |
| Flux | 5 |
| French Pox | 23 |
| Gout | 91 |
| Gravel, Stone, and Stranguary | 11 |
| Grief | 4 |
| Headmouldshot, Horshoe-head, and Water in the Head | 76 |
| Jaundice | 78 |
| Jaw Locked | 1 |
| Imposthume | 1 |
| Inflammation | 433 |
| Itch | 2 |
| Leprosy | 1 |
| Livergrown | 10 |
| Lunatick | 107 |
| Measles | 223 |
| Miscarriage | 3 |
| Mortification | 226 |
| Palpitation of the Heart | 2 |
| Palsy | 105 |
| Pleurisy | 14 |
| Quinsy | 1 |
| Rash | 1 |
| Rheumatism | 3 |
| Scurvy | 3 |
| Small-pox | 1111 |
| Sore Throat | 12 |
| Sores and Ulcers | 11 |
| Spasm | 2 |
| Stoppage in the Stomach | 11 |
| St. Vitus's Dance | 1 |
| Swine Pox | 2 |
| Teeth | 335 |
| Thrush | 35 |
| Worms | 11 |

| | |
|---|---|
| Bit by a Mad Dog | 2 |
| Broken Limbs | 4 |
| Bruised | 2 |
| Burnt | 13 |
| Drowned | 99 |
| Excessive Drinking | 5 |
| Executed * | 12 |
| Found Dead | 10 |
| Fractured | 2 |
| Frighted | 2 |
| Frozen | 2 |
| Killed themselves | 28 |
| Killed by Falls and several other Accidents | 64 |
| Murdered | 3 |
| Poisoned | 6 |
| Scalded | 2 |
| Shot | 1 |
| Smothered | 1 |
| Starved | 4 |
| Suffocated | 7 |
| Total | 269 |
| Christened | |

* There have been executed in Middlesex and Surry 25; of which Number 12 only have been reported to be buried (as such) within the Bills of Mortality.

| | | | |
|---|---|---|---|
| Chriftened | Males - - - | 10087 | In all 18970 |
| | Females - - | 8883 | |
| Buried | Males - - - | 9046 | In all 18134 |
| | Females - - | 9088 | |

Of this number have died,

| | | | |
|---|---|---|---|
| Under Two Years of Age | 5211 | Fifty and Sixty - | 1758 |
| Between Two and Five | 1790 | Sixty and Seventy - | 1565 |
| Five and Ten - - | 644 | Seventy and Eighty - | 1125 |
| Ten and Twenty - | 573 | Eighty and Ninety - | 456 |
| Twenty and Thirty - | 1299 | Ninety and a Hundred - | 63 |
| Thirty and Forty - | 1724 | A Hundred and One - | 2 |
| Forty and Fifty - | 1924 | | |

Decreafed in the Burials this Year 21.

